# Regulation of Banana Phytoene Synthase (MaPSY) Expression, Characterization and Their Modulation under Various Abiotic Stress Conditions

**DOI:** 10.3389/fpls.2017.00462

**Published:** 2017-04-03

**Authors:** Navneet Kaur, Ashutosh Pandey, Shivani, Prateek Kumar, Pankaj Pandey, Atul K. Kesarwani, Shrikant S. Mantri, Praveen Awasthi, Siddharth Tiwari

**Affiliations:** ^1^National Agri-Food Biotechnology Institute (NABI), Department of Biotechnology, Ministry of Science and Technology (Government of India)Mohali, India; ^2^Department of Biotechnology, Panjab UniversityChandigarh, India

**Keywords:** antioxidant activity, banana, carotenoid, gene expression, provitamin A, stress

## Abstract

Phytoene synthase (PSY) is a key regulatory enzyme of carotenoid biosynthesis pathway in plants. The present study examines the role of PSY in carotenogenesis and stress management in banana. Germplasm screening of 10 Indian cultivars showed that Nendran (3011.94 μg/100 g dry weight) and Rasthali (105.35 μg/100 g dry weight) contained the highest and lowest amounts of β-carotene, respectively in ripe fruit-pulp. Nendran ripe pulp also showed significantly higher antioxidant activity as compared to Rasthali. Meta-analysis of three banana *PSY* genes (*MaPSY1, MaPSY2*, and *MaPSY3*) was performed to identify their structural features, subcellular, and chromosomal localization in banana genome. The distinct expression patterns of *MaPSY1, MaPSY2*, and *MaPSY3* genes were observed in various tissues, and fruit developmental stages of these two contrasting cultivars, suggesting differential regulation of the banana *PSY* genes. A positive correlation was observed between the expression of *MaPSY1* and β-carotene accumulation in the ripe fruit-peel and pulp of Nendran. The presence of stress responsive *cis*-regulatory motifs in promoter region of *MaPSY* genes were correlated with the expression pattern during various stress (abscisic acid, methyl jasmonate, salicylic acid and dark) treatments. The positive modulation of *MaPSY1* noticed under abiotic stresses suggested its role in plant physiological functions and defense response. The amino acid sequence analysis of the PSY proteins in contrasting cultivars revealed that all PSY comprises conserved domains related to enzyme activity. Bacterial complementation assay has validated the functional activity of six PSY proteins and among them PSY1 of Nendran (Nen-PSY1) gave the highest activity. These data provide new insights into the regulation of *PSY* expression in banana by developmental and stress related signals that can be explored in the banana improvement programs.

## Introduction

Carotenoids are the natural compounds that are synthesized by vascular plants, some fungi and bacteria, but not by animals including humans (Ruiz-Sola and Rodríguez-Concepción, 2012). Carotenoids are essential for plant growth and development, and human nutrition. They are predominantly synthesized through plastid localized 2-C-methyl-D-erythritol 4-phosphate (MEP) pathway in chloroplasts and chromoplasts (Flores-Perez et al., 2008; Rodriguez-Concepcion, 2010; Jarvis and López-Juez, 2013; Kaur et al., 2016) (Supplementary Figure S1). Chromoplasts can differentiate from the pre-existing chloroplasts during fruit ripening or directly from proplastids, leucoplasts and amyloplasts in non-green tissues to facilitate carotenoid storage (Sandmann et al., 2006; Maass et al., 2009; Jarvis and López-Juez, 2013; Lado et al., 2015).

Phytoene synthase (PSY) regulates carotenoid metabolic flux in the downstream enzymatic steps for the biosynthesis of lycopene, α-carotene and β-carotene (Sandmann et al., 2006; Cazzonelli and Pogson, 2010; Grassi et al., 2013; Fu et al., 2014). Many plant species are known to have multiple copies of *PSY* gene (Gallagher et al., 2004; Clotault et al., 2008; Giorio et al., 2008; Li et al., 2008; Fu et al., 2014; Ampomah-Dwamena et al., 2015). The presence of multiple *PSY* in plants is a result of gene duplication events, which have significance for function and modulation of carotenogenesis (Gallagher et al., 2004; Ampomah-Dwamena et al., 2015). A number of *PSY* genes have been overexpressed in various plants, such as rice (Ye et al., 2000), tobacco (Busch et al., 2002), tomato (Fraser et al., 2002) and arabidopsis (Maass et al., 2009) leading to enhanced biosynthesis of carotenoids in a specific tissue.

Carotenoids play protective roles against biotic/abiotic stresses in plants. They are involved in signaling process and scavenging of free radicals during the stress management (El-Agamey et al., 2004; Aquino et al., 2016; García-Ruiz et al., 2017). Oxidized carotenoids (apocarotenoids) lead to produce abscisic acid (ABA), strigolactone, β–cyclocitral and β-ionone which subsequently assist plants to adapt in different stress conditions (Pogson et al., 2008; Havaux, 2014; Hou et al., 2016).

Abscisic acid (ABA), salicylic acid (SA), and jasmonic acid (JA) are the major phytohormones which play important roles in the regulation of plant growth and development, and management of variety of abiotic (water, cold, desiccation, and salinity), and biotic (pathogen) stresses (Clarke et al., 2009; Brossa et al., 2011; Rivas et al., 2011; Wang et al., 2011; Khan et al., 2015). They are also key factors in signaling pathway as well as important regulators of defense gene expression and immune response mechanisms (Adie et al., 2007; Koornneef and Pieterse, 2008; Solano and Gimenez-Ibanez, 2013). The slight change in ABA concentration can alter the antioxidative defense mechanism which lead to variation in oxidative damage intensity (Jiang and Zhang, 2001; Nayyar et al., 2005; Nayyar and Gupta, 2006; Duan et al., 2007; Rivas et al., 2011). The *PSY* is reported for feedback regulation of ABA biosynthesis (Welsch et al., 2008). Stress tolerance conferred by *PSY* alleles through regulation of ABA in rice, maize and cassava suggested their role in stress control mechanism (Li et al., 2008; Welsch et al., 2008; Arango et al., 2010). The β–cyclocitral produces by oxidation of the β-carotene has role in SA accumulation during the excess light stress (Lv et al., 2015). SA regulates the plant physiological functions and defense mechanism against abiotic stresses (Loake and Grant, 2007; Bari and Jones, 2009; Vlot et al., 2009; An and Mou, 2011). JA and its derivatives such as methyl jasmonate (MeJA) are reported to be involved in various plant biological activities including cell division, fruit growth and ripening (Creelman and Mullet, 1997). It also plays an important role in plant adaptation during abiotic stresses (Clarke et al., 2009; Yoon et al., 2009; Brossa et al., 2011; Nitschke et al., 2016).

Herein, we identified two contrasting cultivars of Indian banana i.e., Nendran and Rasthali with different levels of β-carotene in their fruit-pulp. The *PSY* was selected as a target gene for finding any correlation with their expression and accumulation of β-carotene in ripe fruit-pulp of contrasting cultivars. The present study also highlighted the differential expression patterns of three banana *PSY* (*MaPSY1, MaPSY2*, and *MaPSY3*) genes in different tissues and during various stress conditions.

## Materials and methods

### Plant materials, experimental conditions and reagents

Ten banana accessions were selected from the field germplasm plot at the National Agri-Food Biotechnology Institute (NABI), Mohali for the carotenoids estimation in ripe fruit-pulp (Table 1). The fruit sampling was performed during the summer season. Plant tissues such as pseudostem (outer, and inner layers), leaf (young, and mature stages), bract, fruit finger, fruit-peel and -pulp (unripe, and ripe stages) were collected from the contrasting cultivars, frozen in liquid nitrogen, and stored at −80°C until further use. For abiotic stress treatments, *in-vitro* grown Rasthali plants were exposed with ABA (100 μM), MeJA (250 μM) and SA (250 μM), as described previously (Sreedharan et al., 2012; Pandey et al., 2016). The control and treated plants were grown in plant growth chambers (Conviron, Canada) at 26°C under 16/8-h light (200 μmol photon m^−2^ s^−1^)/dark period with 60 ± 5% relative humidity (RH). To study the effect of dark condition, plants were kept under the dark for 72 h in a plant growth chamber (Conviron, Canada) at 26°C with 60 ± 5% RH. Leaves were collected at different time intervals, frozen immediately in the liquid nitrogen, and stored at −80°C till further use. All biochemical, standards, kits and reagents unless otherwise stated were molecular biology or cell culture grade from Sigma Chemical Company (St. Louis, MO) and Merck (India).

**Table 1:** **Carotenoids (μg/100g DW) in ripe fruit-pulp of Indian banana cultivars**.

**S.No**.	**Musa cultivars**	**Genome**	**Fruit type**	**β-Carotene**	**α-Carotene**	**β-Carotene equivalents^a^**	**Lutein**	**Zeaxanthin**	**Lycopene**
1	Ney Poovan	AB	Dessert	166.56 ± 8.56	111.96 ± 2.32	222.54	272.27 ± 8.65	ND	ND
2	Grand Naine	AAA	Dessert	422.66 ± 90.02^*^	215.66 ± 16.45^**^	530.49	176.00 ± 17.64	ND	ND
3	Red Banana	AAA	Dessert	438.00 ± 14.00^*^	243.00 ± 19.00^**^	559.50	2319.00 ± 186.72^**^	ND	ND
4	Manoranjitham	AAA	Dessert	179.81 ± 16.62	54.50 ± 5.50	207.06	69.50 ± 8.50	ND	ND
5	Robusta	AAA	Dessert	192.00 ± 13.00	82.50 ± 6.27	233.25	249.92 ± 9.02	ND	ND
6	Rasthali	AAB	Dessert	105.35 ± 7.35	129.40 ± 4.00	170.05	377.04 ± 41.10	ND	ND
7	Nendran	AAB	Cooking	3011.94 ± 176.70^**^	1377.34 ± 325.77^**^	3700.58	182.55 ± 13.20	ND	ND
8	Poovan	AAB	Dessert	930.62 ± 38.86^**^	278.26 ± 57.43^**^	1069.75	570.57 ± 123.12	ND	ND
9	KachaKola	ABB	Cooking	171.84 ± 14.52	112.81 ± 2.84	228.25	222.00 ± 13.54	ND	ND
10	Karpuravalli	ABB	Dessert	134.02 ± 12.35	16.31 ± 3.48	142.18	162.76 ± 28.00	ND	ND

a *β-Carotene Equivalents calculated by the sum of β-carotene plus one-half of α-carotene*.

Values represent mean of carotenoid content ± SD. Prism GraphPad software (GraphPad Software Inc., San Diego, CA, USA) was used to compare the mean by Student's paired t-test. Statistical significant was checked at p ≤ 0.05 - 0.01 (

* *p ≤ 0.05*,

** *p ≤ 0.01) with respect to the Rasthali. The values without asterisk marked are not significantly different*.

*DW, Dry weight; ND, Not detected*.

### Quantitative estimation of carotenoids

Carotenoid extraction was performed as described previously (Matsumoto et al., 2007; Flowerika et al., 2016) with some modifications. The lyophilized 1 g dry weight (DW) sample was homogenized in 50 ml solution containing 40% (v/v) methanol + 0.5 g magnesium carbonate and centrifuged. The insoluble fraction was extracted in diethyl ether and methanol (7:3, v/v) containing 0.1% (w/v) butylated hydroxy toluene (BHT) till the extract turned colorless. The extract was transferred in a separating funnel and 20 ml diethyl ether was added. The ether phase was washed three-times with 10 ml of saturated sodium chloride and 5 ml of 10% anhydrous sodium sulfate solution. The sample was evaporated using Rota vapor (Buchi Labortechnik AG) at 50°C at 100 rpm and 200 atmospheric pressures. The residue was dissolved in methyl tert-butyl ether (MTBE)-methanol (1:1, v/v) containing 0.1% (w/v) BHT, and centrifuged. The supernatant was filtered through 0.45-μm nylon membrane filter (Millipore, MA) and was used for High Performance Liquid Chromatography (HPLC). Analysis was performed in a liquid chromatograph binary gradient module pumps (Waters, Milford, MA, USA) equipped with photodiode array (Waters, 2998) and autosampler (Waters, 2767). Carotenoids were separated by using YMC C_30_ column (YMC, Kyoto, Japan). The mobile phase was a gradient, prepared from 95% (v/v) methanol in HPLC-grade water (component A) and MTBE (component B). Carotenoid standards (β-carotene, lutein, zeaxanthin and lycopene) were used for calibration curve, comparison of retention time, and quantification of samples. Data was analyzed using MassLynx™ software and quantification was carried out at 450 nm.

### Measurement of antioxidant activity

To determine antioxidant activity, carotenoid extract was prepared from pulp tissue of Rasthali and Nendran as per procedure described above. Measurement of radical scavenging activity was carried out as per earlier reported 2,2-diphenyl-1-picrylhydrazyl (DPPH) method (Blois, 1958; Awasthi et al., 2016). Briefly, freshly prepared DPPH methanolic solution was added to 200 μl of methanol having different concentration of carotenoid extract. Reaction was incubated in dark for 30 min at room temperature. The absorbance was measured at 517 nm (UV-2700 UV-VIS Spectrophotometer, Shimadzu). Percent inhibition was calculated according to the following equation:

$$\%\text{ Inhibition}=\left[\left({\text{Ab}}_{{\text{DPPH}}^{*}}-{\text{Ab}}_{{\text{S}}^{**}}\right)/{\text{Ab}}_{{\text{DPPH}}^{*}}\right]\times \text{100},$$

^*^Ab_DPPH_: Absorbance of DPPH solution at 517 nm,
    ^**^Ab_S_: Absorbance of DPPH solution containing extract at a particular concentration at 517 nm.

Experiment was performed in three replicates and ascorbic acid was used as positive control. IC_50_ values (half maximal inhibitory concentration) were calculated as the concentration of extracts causing 50% inhibition of DPPH radical. Lower IC_50_ value corresponds to a higher antioxidant activity of extract.

### Identification and sequence analysis of *MaPSY*

The whole genome sequence of banana (*Musa acuminata*) was downloaded from the Banana Genome Hub database (D'Hont et al., 2012; http://banana-genome.cirad.fr/) and used for the identification of *PSY* gene. BLAST search was performed using annotated *PSY* sequences of maize (*Zea mays*), rice (*Oryza sativa*) and arabidopsis (*Arabidopsis thaliana*) to explore similar sequences in the banana genome database. The reported *PSY* gene sequences of banana were also considered in BLAST analysis. The most similar sequences were retrieved from the banana genome database and designated as putative *MaPSY* genes. The percentage identity of the predicted protein sequences was noted. Selected coding (CDS) and genomic DNA sequences were aligned to identify the gene structures, and numbers of exons and introns in the sequences. Theoretical isoelectric point and molecular weight were predicted using the Compute pI/MW tool on the ExPASy server (Bjellqvist et al., 1994; http://web.expasy.org/compute_pi/). Transmembrane domains were analyzed on the TMHMM server v 2.0 (Krogh et al., 2001; http://www.cbs.dtu.dk/services/TMHMM-2.0/) and subcellular localization of deduced proteins predicted on the TargetP 1.1 server (Emanuelsson et al., 2007; http://www.cbs.dtu.dk/services/TargetP/). The presence of chloroplast transit peptides was predicted using the ChloroP 1.1 server (Emanuelsson et al., 1999). Mapping of the biosynthetic genes on banana chromosome was done by using the locus ID information available on Banana Genome Hub (http://banana-genome.cirad.fr/). The upstream regions (1.5 kbp) of all selected genes were extracted through a custom Perl script for the identification of *cis*-regulatory elements. This region was considered as the proximal promoter for the individual gene. The promoter sequences were placed in the Plant Care Database (Lescot et al., 2002) in which a brief description of all motifs were extracted and presented.

### RNA isolation, cDNA preparation, and expression analysis

Total RNA was isolated from different tissues (pseudostem, leaf, bract, fruit finger, fruit-peel, and -pulp), and treated samples (ABA, MeJA, SA, and dark) according to previously described protocol (Asif et al., 2006). RNA sample was treated with DNase I Digest kit (Sigma-Aldrich, USA) to eliminate DNA contamination. The cDNA synthesis was performed using Revert Aid First Strand cDNA synthesis Kit (Fermentas, USA) from 3 μg of DNA-free total RNA following the manufacturer's instructions.

The quantitative real-time PCR expression was carried out by following SYBR Green chemistry at ABI PRISM 7700 Fast Real-Time PCR System (Applied Biosystems, USA). The invariant expression under the described experimental conditions was normalized by using two banana housekeeping genes, *actin1* (GenBank Accession No. AF246288) and *ubiquitin2* (GenBank Accession No. HQ853254). The relative fold expression was calculated by using 2^−ΔΔCT^ method (Schmittgen and Livak, 2008). The list of different primers used in the study is given in Supplementary Table S1.

### Cloning, protein sequence and phylogenetic analyses

Three *MaPSY* sequences retrieved from *Musa acuminata* reference genome were used for designing the gene specific primers (Supplementary Table S1). The full-length open reading frames (ORFs) of *PSY* were amplified from the ripe fruit-pulp of contrasting cultivars (Nendran and Rasthali). The genes isolated from Nendran and Rasthali are designated as *NEN-PSY1, NEN-PSY2, NEN-PSY3*, and *RAS-PSY1, RAS-PSY2, RAS-PSY3* respectively. The amplicons containing *Bgl*II and *Not*I restriction sites were cloned in pBluescript SK+ vector (Stratagene, USA) and confirmed by sequencing. The conserved motifs and sites for PSY functional activity were determined by using NCBI CD-Search (Marchler-Bauer et al., 2011; http://www.ncbi.nlm.nih.gov/Structure/bwrpsb/bwrpsb.cgi) and ScanProsite (Hulo et al., 2008; http://prosite.expasy.org/scanprosite) tools. PSY protein sequence annotation analysis was accelerated using BioEdit sequence alignment editor (Hall, 1999; http://www.mbio.ncsu.edu/bioedit/bioedit). Phylogenetic analysis was conducted in MEGA6 (Tamura et al., 2013) for deduced protein sequences of arabidopsis, rice, maize and banana. The PSY sequences derived from Asupina (high provitamin A) and Cavendish (low provitamin A) banana cultivars (Mlalazi et al., 2012) were also considered in the study. Neighbor-joining method was used to infer homologs and evolution. Bootstrap value was set to 1000.

### Functional complementation analysis of six MaPSY in *E. coli*

A heterologous bacterial complementation system was used to investigate the biological function of six MaPSY proteins derived from Nendran (NEN-PSY1, NEN-PSY2, NEN-PSY3), and Rasthali (RAS-PSY1, RAS-PSY2, and RAS-PSY3). The plasmids pAC-85b and pTrc were used to test the functional activity of *PSY* genes (Cunningham and Gantt, 2007; Fu et al., 2014). The *E. coli* cells transformed with these plasmids could not be able to synthesize carotenoid which resulted white colonies. The PCR products of six *MaPSY* genes comprising *Bgl*II and *Not*I restriction sites (Supplementary Table S1) were cloned into digested pTrc plasmid and named as pTrc-NEN-PSY1, pTrc-NEN-PSY2, pTrc-NEN-PSY3, pTrc-RAS-PSY1, pTrc-RAS-PSY2, and pTrc-RAS-PSY3. The competent *E. coli* cells were co-transformed with pAC-85b and pTrc plasmid having *PSY* genes. A single positive colony was inoculated in Luria Broth (LB) medium containing ampicillin (100 μg/ml) and chloramphenicol (50 μg/ml) antibiotics and grown at 37°C with 200 rpm. The relative activity of MaPSY proteins could be observed as variation in the color of cultures due to differential accumulation of carotenoid. The β-carotene was extracted from the bacterial cell pellets (0.6 OD) and analyzed by HPLC by the method described earlier (Matsumoto et al., 2007; Flowerika et al., 2016). The extractions were repeated at least three times from independent transformed bacterial cell cultures.

### Statistical analysis

All experiments were repeated with three biological replicates and each experiment consisted of three technical replicates. The data are presented as mean ± SD and analyzed by Student's paired *t*-test. The mean values under each treatment were compared to determine significance (^*^P ≤ 0.05; ^**^P ≤ 0.001; ^***^P ≤ 0.0001). The relationship between gene expression and individual carotenoid accumulation was calculated by Pearson correlation (r) analysis. *R*^2^ values were considered significant (^*^P ≤ 0.05; ^**^P ≤ 0.005; ^***^P ≤ 0.001) correlations. All statistical analyses were performed using Prism GraphPad software Ver. 5.01 (GraphPad Software Inc., San Diego, CA, USA).

## Results

### Carotenoid profiling during banana fruit development

**Figure 1:**
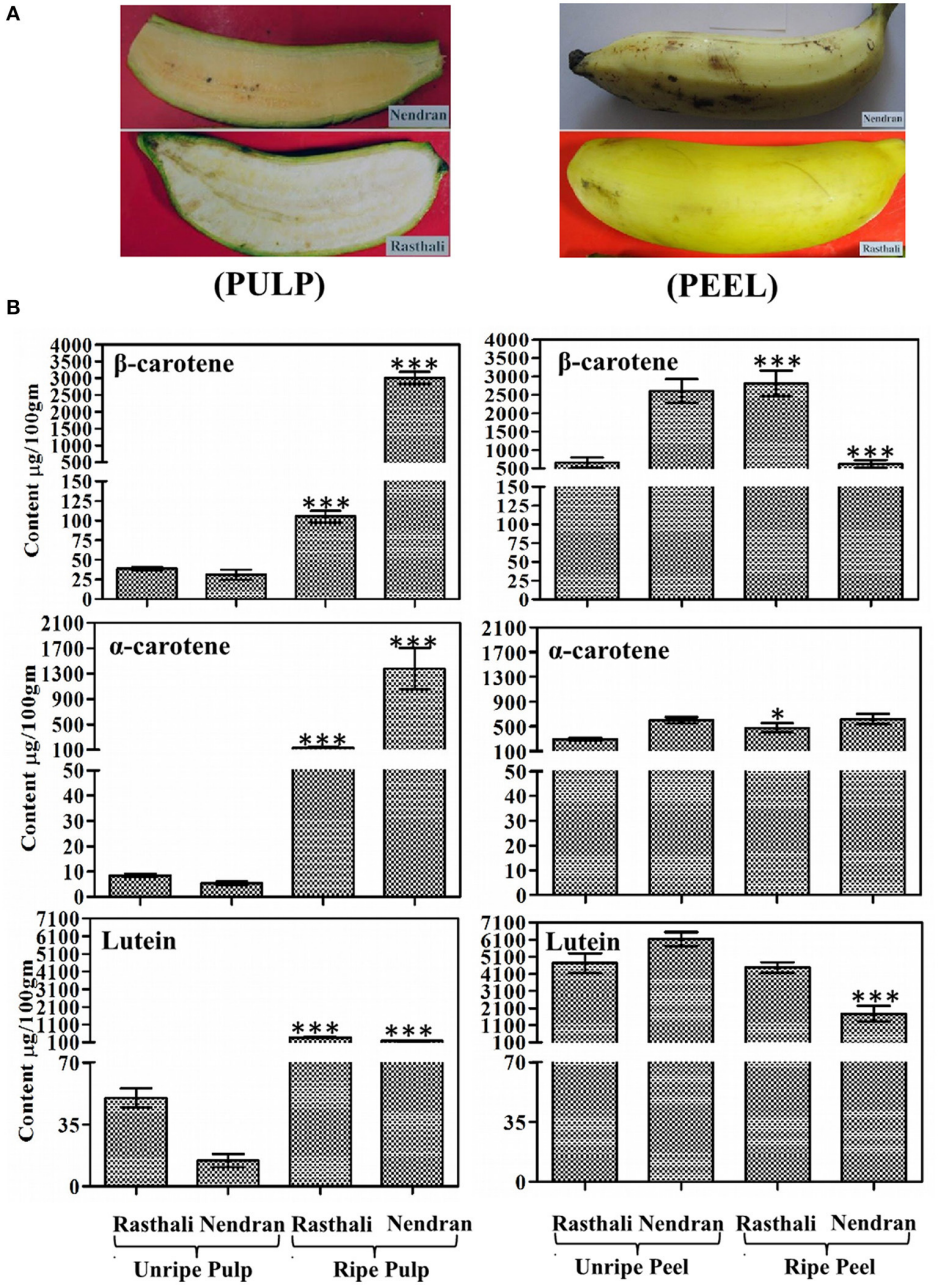
**Qualitative and quantitative assessment of carotenoids in fruit tissues of Nendran and Rasthali banana cultivars**. **(A)** Visual observation of ripe fruit-pulp and -peel. **(B)** Carotenoid profiling in ripe and unripe fruit tissues. Bars denote mean of carotenoid content ± SD. Compounds were quantified by separating extracts from the peel and pulp using HPLC. HPLC chromatograms are given in Supplementary Figures S2, S3. Statistical analysis was performed using Student's paired *t*-test. Statistical significance was checked at *P* ≤ 0.05–0.001 (^*^P ≤ 0.05; ^***^P ≤ 0.0001) with respect to the unripe- versus ripe-pulp/peel of Rasthali and Nendran.

Fruit-pulp collected at the ripening stage of 10 Indian banana cultivars were lyophilized and analyzed for carotenoids. The highest β-Carotene Equivalents (β-carotene + ½ α-carotene) was observed in Nendran (3700.57 μg/100 g DW) while the lowest was found in Rasthali (170.05 μg/100 g DW) (Table 1). The ripe pulp of Rasthali appeared creamy-white and Nendran was deep-yellow in color (Figure 1A). Nendran and Rasthali cultivars were studied for changes in carotenoids in fruit-peel and -pulp as collected at unripe and ripe stages (Figure 1B). Lutein, α-carotene and β-carotene were quantified, whereas lycopene and zeaxanthin were not present in detectable amounts in the tissues. In the pulp tissue, the higher content of all the carotenoids was noticed at the ripening stage. Nearly 28-fold higher β-carotene was present in Nendran (3011.94 ± 176.70 μg/100 g DW) as compared to Rasthali (105.35 ± 7.35 μg/100 g DW) (Figure 1B). Similarly, α-carotene was nearly 10-fold higher in Nendran as compared to Rasthali. Lutein was nearly 2-fold higher in Rasthali as compared to Nendran. Unripe pulp of both Nendran and Rasthali showed significantly low quantities of all carotenoids, compared to the ripe pulp (Figure 1B, Supplementary Figure S2). However, in the peel as compared to the pulp of both the cultivars, higher deposition of all carotenoids (except at the ripening stage of Nendran) was noticed (Figure 1B, Supplementary Figure S3). The greater deposition of α-carotene and β-carotene was found in ripe pulp of Nendran as compared to its ripe peel (Figure 1B).

### Antioxidant activity

In the present study, Nendran unripe/ripe pulp extracts showed the lower IC_50_ value in comparison to Rasthali unripe/ripe extract (**Table 4**). Ripe pulp extract of both Nendran and Rasthali had lower IC_50_ value as compared to the unripe pulp extract. The IC_50_ values for the extracts of unripe and ripe pulp of Nendran and Rasthali are given in **Table 4**. The IC_50_ value of all the extracts was lower than that of ascorbic acid (5.66 ± 0.86), which was used as positive control in the experiment.

### *In-silico* analysis of *PSY* in banana

Three homologs of *PSY* (*MaPSY1*-*3*) were identified in Banana Genome Hub database using known proteins from arabidopsis, maize and rice. Percentage identity of the predicted protein sequences of the banana *PSY* genes showed high homology with those in arabidopsis, maize and rice varies from 55 to 80% (Supplementary Table S2). Structural analysis of exons and introns of *PSY* from banana and other species is presented in Supplementary Table S3. The *PSY* homologs from banana, maize and rice had same number of exons (6) and introns (5) however, arabidopsis *PSY* was found to be without intron. The accession numbers of the genes, their positions on banana chromosome, predicted polypeptide length, isoelectric point, molecular mass, subcellular localization and transmembrane helices (TMH) are given in Supplementary Table S4. *MaPSY1* and *MaPSY2* were located on the chromosomes 6 and 9, respectively, while *MaPSY3* was present on the un_random chromosome (uncharacterized scaffold). The MaPSY1 and MaPSY2 proteins had similar length of 397 amino acids whereas MaPSY3 was larger in length and contained 426 amino acids. The isoelectric point (9.38), and molecular weight (47.76 kDa) of MaPSY3 was higher than MaPSY1 (9.22, and 45.03 kDa), followed by MaPSY2 (9.12, and 44.35 kDa). All the MaPSY showed to be localized in chloroplast. MaPSY1 has shown the presence of transmembrane helix.

### Gene expression in different tissues

**Figure 2:**
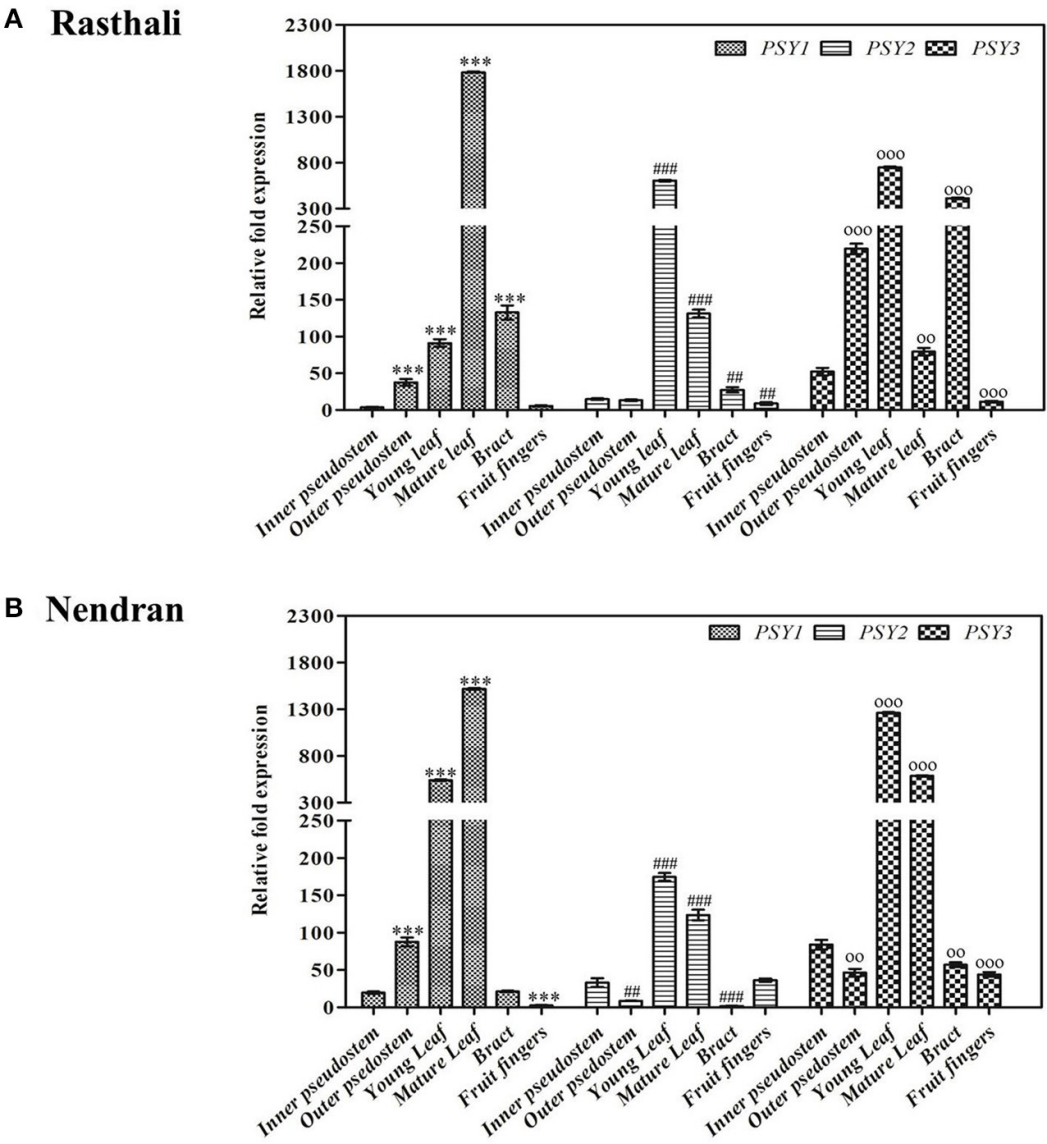
**Spatiotemporal real-time PCR expression analysis of ***PSY*** genes in banana vegetative tissues**. Transcript expression profiles are presented in pseudostem (inner, and outer), leaf developmental stages (young, and mature), bract, and fruit fingers of Rasthali **(A)**, and Nendran **(B)**. The gene expression was normalized with reference to *actin1* taken as internal control. Bars denote mean fold expression as compared to the lowest expressing gene in the group of samples ± SD. Statistical analysis was performed using Student's paired *t*-test. Statistical significance was checked at *P* ≤ 0.05–0.001 and the symbols on the top of bars represents significance levels (##, oo, *P* ≤ 0.001; and ^***^, ###, ooo, *P* ≤ 0.0001) with respect to inner pseudostem in each group.

**Figure 3:**
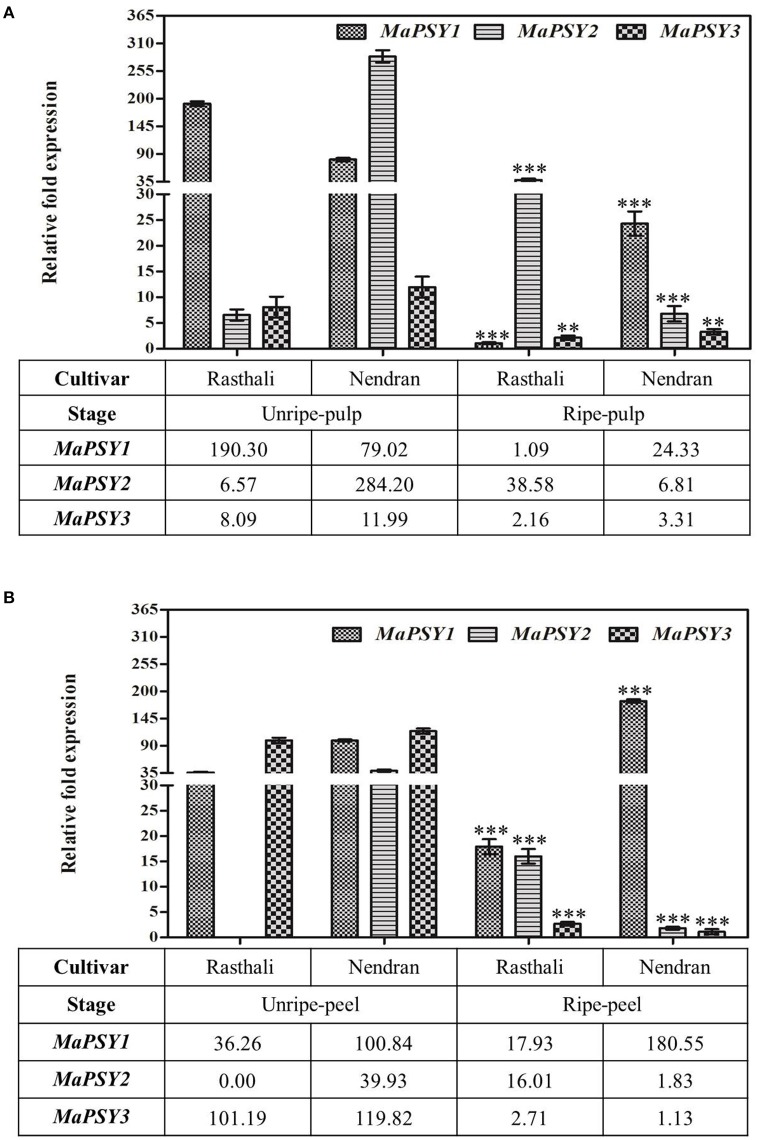
**Spatiotemporal real-time PCR expression analysis of ***PSY*** genes in banana fruit tissues**. Transcript expression profiles are presented in unripe/ripe -pulp **(A)** and -peel **(B)** of Rasthali and Nendran cultivars. The gene expression was normalized with reference to *actin1* taken as internal control. Bars denote mean fold expression as compared to the lowest expressing gene in the group of samples ± SD. Statistical analysis was performed using Student's paired *t*-test. Statistical significance was checked at *P* ≤ 0.05–0.001 (^**^P ≤ 0.001; ^***^P ≤ 0.0001) with respect to the unripe- versus ripe-pulp/peel of Rasthali and Nendran.

Expression analysis of three *PSY* homologs (*MaPSY1, MaPSY2*, and *MaPSY3*) in different tissues of contrasting cultivars (Rasthali and Nendran) was performed (Figures 2, 3). The normalization of transcripts was carried out against *actin1* gene that showed most stable expression in various tissues. In general, the expression pattern of *MaPSY* homologs was similar in both cultivars, but they expressed at varying levels in different tissues. In the pseudostem of both cultivars, higher expression of *MaPSY3* was detected in inner and outer layer (Figures 2A,B). The lower expression of *MaPSY1* and *MaPSY2* was observed in inner and outer layer of both cultivars, respectively. Two leaf developmental stages i.e., young and mature leaves were taken for the analysis (Figures 2A,B). The higher transcript level of *MaPSY3* and *MaPSY1* was noted in young leaf of Nendran and Rasthali, respectively. Mature leaf of both cultivars has shown higher expression of *MaPSY1*. The lower expression of *MaPSY2* was observed in young and mature leaf of both cultivars. Bract and fruit fingers of both cultivars showed higher expression of *MaPSY3*. The lower expression of *MaPSY2* and *MaPSY1* was noted in bracts and fruit fingers of both cultivars, respectively (Figures 2A,B). These results suggest differential expression of *MaPSY* genes among different tissues as well as between the contrasting cultivars. The analysis indicated that all *MaPSY* gene homologs were highly expressed in leaf tissue of both cultivars (Figures 2A,B).

To understand the transcriptional regulation in the carotenogenesis in fruit tissues of two contrasting cultivars, expression of *MaPSY* homologs was studied in fruit-pulp (Figure 3A) and -peel (Figure 3B) at two developmental stages (unripe and ripe). The homologs were expressed in all fruit tissues of both cultivars, except *MaPSY2*. The transcript of *MaPSY2* was not detected in unripe peel of Rasthali. In the unripe pulp, higher expression of *MaPSY1* and *MaPSY2* was noticed in Rasthali and Nendran, respectively. Whereas in ripe pulp, high transcript level of *MaPSY2* and *MaPSY1* was observed in Rasthali and Nendran, respectively (Figure 3A). In the peel at unripe stage, higher expression of *MaPSY3* was recorded in Rasthali as well as in Nendran (Figure 3B). The higher expression of *MaPSY1* was observed at the ripe stage of peel in both cultivars.

### Relationship between *MaPSY* expression and carotenoid accumulation in fruit tissues

Pearson correlation analysis was carried out between relative fold expression of *MaPSY* transcripts and individual carotenoid content in fruit-pulp (Table 2) and -peel (Table 3) in the contrasting cultivars. In the Nendran pulp, significant positive correlation was observed for *NEN-PSY1* (*r* = 0.9995, *P* < 0.05) and *NEN-PSY2* (*r* = 0.9978, *P* < 0.05) with β-carotene and α-carotene, respectively. No significant correlation was noted for lutein in Nendran pulp. In Rasthali pulp, significant positive correlation was noted for *RAS-PSY2* (*r* = 1.00, *P* < 0.005) and *RAS-PSY1* (*r* = 0.9973, *P* < 0.05) with β-carotene. *RAS-PSY2* (*r* = 0.9969, *P* < 0.05) showed significant positive correlation with α-carotene. Lutein accumulation in Rasthali showed significant positive correlation with both *RAS-PSY1* (*r* = 0.9983, *P* < 0.05) and *RAS-PSY2* (*r* = 0.9998, *P* < 0.05). In the Nendran peel, significant positive correlation was established for *NEN-PSY1* (*r* = 1.00, *P* < 0.005) with β-carotene whereas, negative correlation was observed for *NEN-PSY1* (*r* = −0.9997, *P* < 0.05) with lutein. No correlation was observed for α-carotene in Nendran. In the Rasthali peel, significant positive correlation was established for *RAS-PSY1* with lutein (*r* = 0.9994, *P* < 0.05). However, with β-carotene significant negative correlation was noted for *RAS-PSY2* (*r* = −0.999, *P* < 0.05). Besides, no significant correlation (*r* = 0.8262 to 0.9945, *P* ≥ 0.1031 to ≤ 0.381) was found between carotenoids and *PSY3* homolog of both cultivars in pulp and peel (Table 2).

**Table 2:** **Correlation of ***PSY*** expression with the accumulation of individual carotenoids in fruit-pulp of Nendran and Rasthali**.

**Genes**	**Nendran lutein (**μ**g/100g DW)**			**Rasthali lutein (**μ**g/100g DW)**		
	**Pearson r**	***P*-value**	***R^2^***	**Pearson r**	***P*-value**	***R^2^***
*PSY1*	0.7834^ns^	0.4269	0.6138	0.9983^^*^^	0.0369	0.9966
*PSY2*	0.932^ns^	0.2361	0.8686	0.9998^^*^^	0.014	0.9995
*PSY3*	0.8262^ns^	0.381	0.6826	0.9692^ns^	0.1584	0.9394
**Genes**	**Nendran** α**-carotene (**μ**g/100g DW)**			**Rasthali** α**-carotene (**μ**g/100g DW)**		
	**Pearson r**	***P*****-value**	***R**^2^*	**Pearson r**	***P*****-value**	***R**^2^*
*PSY1*	0.9727^ns^	0.1490	0.9462	0.9875^ns^	0.1008	0.9751
*PSY2*	0.9978^^*^^	0.0418	0.9957	0.9969^^*^^	0.0499	0.9939
*PSY3*	0.9869^ns^	0.1031	0.9740	0.9397^ns^	0.2222	0.883
**Genes**	**Nendran** β**-carotene (**μ**g/100g DW)**			**Rasthali** β**-carotene (**μ**g/100g DW)**		
	**Pearson r**	***P*****-value**	***R**^2^*	**Pearson r**	***P*****-value**	***R**^2^*
*PSY1*	0.9995^^*^^	0.0209	0.9989	0.9973^^*^^	0.0471	0.9945
*PSY2*	0.9452^ns^	0.2117	0.8935	1.00^^**^^	0.0038	1.00
*PSY3*	0.9945^ns^	0.0668	0.989	0.965^ns^	0.1685	0.9316

ns , not significant;

* , significant at P < 0.05;

** *, highly significant at P < 0.005*.

**Table 3:** **Correlation of ***PSY*** expression with the accumulation of individual carotenoids in fruit-peel of Nendran and Rasthali**.

**Genes**	**Nendran lutein (**μ**g/100g DW)**			**Rasthali lutein (**μ**g/100g DW)**		
	**Pearson r**	***P*-value**	***R^2^***	**Pearson r**	***P*-value**	***R^2^***
*PSY1*	−0.9997^^*^^	0.0164	0.9993	0.9994^^*^^	0.0224	0.9988
*PSY2*	−0.869^ns^	0.3302	0.7543	0.9868^ns^	0.1034	0.9738
*PSY3*	−0.8755^ns^	0.321	0.7666	0.9715^ns^	0.1523	0.9439
**Genes**	**Nendran** α**-carotene (**μ**g/100g DW)**			**Rasthali** α**-carotene (**μ**g/100g DW)**		
	**Pearson r**	***P*****-value**	***R**^2^*	**Pearson r**	***P*****-value**	***R**^2^*
*PSY1*	0.9255^ns^	0.2473	0.8566	0.9551^ns^	0.1915	0.9123
*PSY2*	0.9945^ns^	0.0665	0.9891	0.8784^ns^	0.3173	0.7715
*PSY3*	0.9959^ns^	0.0573	0.9919	0.9953^ns^	0.0616	0.9907
**Genes**	**Nendran** β**-carotene (**μ**g/100g DW)**			**Rasthali** β**-carotene (**μ**g/100g DW)**		
	**Pearson r**	***P*****-value**	***R**^2^*	**Pearson r**	***P*****-value**	***R**^2^*
*PSY1*	1.0000^^**^^	0.0043	1.0000	−0.987^ns^	0.1019	0.9746
*PSY2*	0.9298^ns^	0.24	0.8645	−0.999^^*^^	0.0239	0.9986
*PSY3*	0.935^ns^	0.2308	0.8742	−0.934^ns^	0.2318	0.8732

ns , not significant;

* , significant at P < 0.05;

** *, highly significant at P < 0.005*.

### Promoter analysis and *MaPSY* expression under stress conditions

Various *cis*-regulatory elements were detected in the proximal promoter regions of three *MaPSY* genes (Supplementary Table S5). The *cis-*elements such as Skn-1 motif related to growth and development, Box4, and Sp1 motifs related to light response were present in all three *MaPSY* genes. The light responsive elements were mostly found in *MaPSY1* and *MaPSY3* while higher number stress responsive motifs were detected in *MaPSY1* and *MaPSY2*, which suggested their possible role in these conditions.

**Figure 4:**
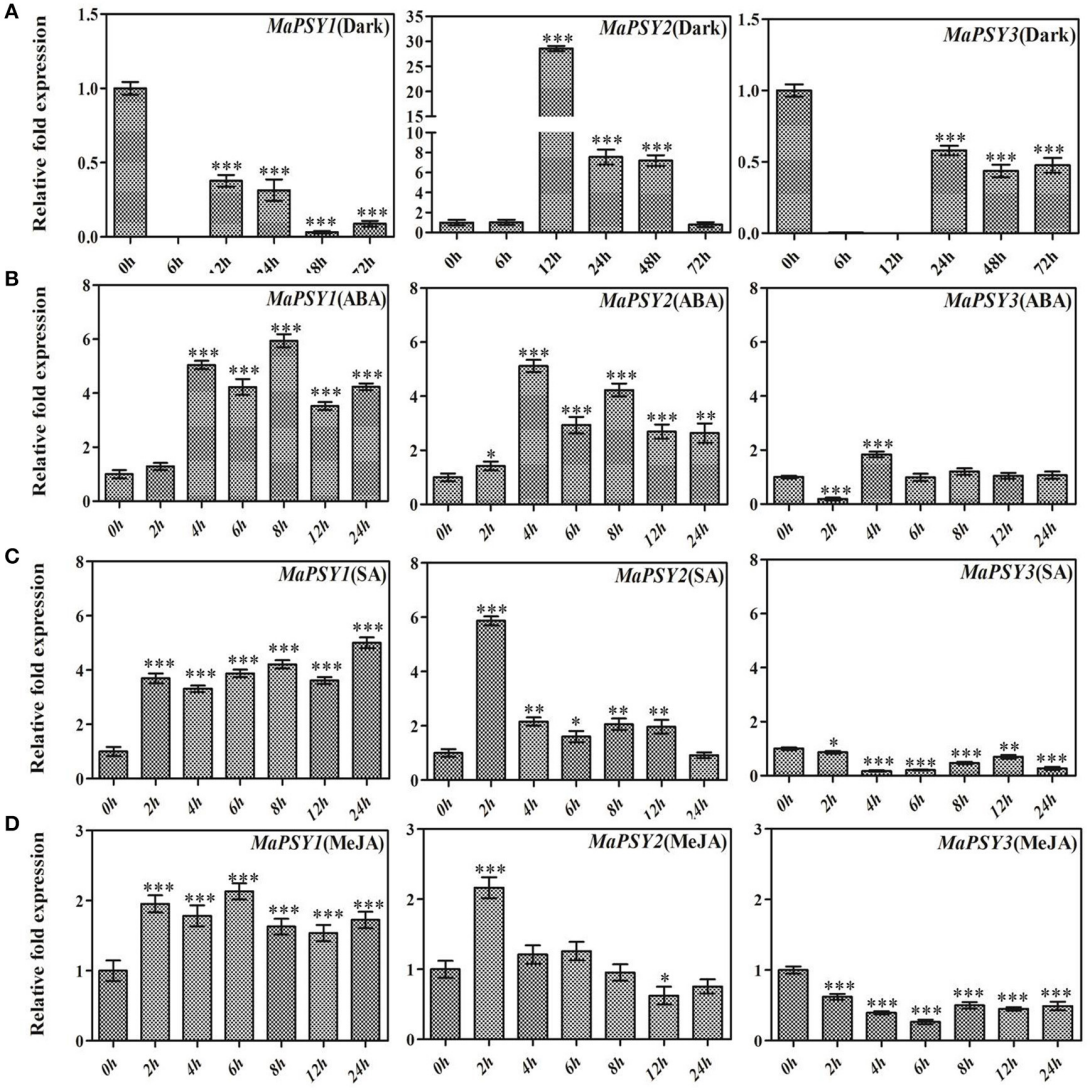
**Real-time PCR expression analysis of ***PSY*** genes under stress conditions**. Transcript expression profiles are presented in *in-vitro* grown Rasthali plants under **(A)** dark, **(B)** methyl jasmonate, **(C)** abscisic acid, and **(D)** salicylic acid exposures. The gene expression was normalized with reference to *actin1* taken as internal control. Bars denote mean fold expression as compared to the control (0 h sample). Statistical analysis was performed using Student's paired *t*-test. Statistical significance was checked at *P* ≤ 0.05–0.001 (^*^P ≤ 0.05; ^**^P ≤ 0.001; ^***^P ≤ 0.0001) with respect to the 0 h sample.

To understand the effect of various environmental factors on transcriptional regulation of *MaPSY* genes, expression analysis was performed following different treatments in Rasthali plants. The differential expression of all three *MaPSY* genes was observed during the dark exposure to the plants. The analysis reveals that the dark exposure leads to significantly down-regulated expression of *MaPSY1* and *MaPSY3* genes (Figure 4A). Interestingly, nearly 26- and 7.5- fold up-regulated expression of *MaPSY2* was noted after 12 and 48 h, respectively of incubation under the dark as compared to control (0 h) plants (Figure 4A). The expression of *MaPSY2* was comparable to control (0 h) at 72 h. The expression patterns suggest a positive correlation with the presence of high number light-responsive *cis-*elements in *MaPSY1* and *MaPSY3* promoter regions.

The exogenous applications of ABA, SA and MeJA have modulated expression of *MaPSY* genes (Figures 4B–D). ABA exposure led to the significant enhancement in the expression of all three *MaPSY* genes at 4 h, but the differential modulation was noted for different genes at different time points (Figure 4B). *MaPSY1* and *MaPSY2* showed a significant up-regulation in the expression at 4 h which continued till 72 h. In case of *MaPSY3*, biphasic expression pattern was observed (Figure 4B). ABRE motif responsible for ABA response was solely present in the promoter region of *MaPSY2* gene.

Exposure of SA led to the significant up-regulation in expression of *MaPSY1* at 2 h and continued till 24 h. On the other hand, *MaPSY2* transcript was found to be up-regulated at 2 h and then reduced to comparable levels of control (Figure 4C). *MaPSY*3 showed significant down-regulation in expression at different time points after treatment. Interestingly, TCA element that is responsible for SA response has found in three copies at the proximal promoter region of *MaPSY1* whereas it was absent in *MaPSY2* promoter. A single copy of TCA element was present in *MaPSY3* promoter region.

The exogenous exposure of MeJA showed nearly 2-fold up-regulation in expression of *MaPSY1* at 2 h which continued till 24 h (Figure 4D). *MaPSY2* expression was up-regulated at 2 h and afterwards expression was comparable to the control (Figure 4D). The significant down-regulation in expression of *MaPSY*3 was found at different time points as compare to control. MeJA responsive elements (CGTCA and TGACG motifs) were present in the proximal promoter regions of *MaPSY1* and *MaPSY2*, but absent in *MaPSY3*. These results suggest that *MaPSY* genes expression in response to different treatments largely correlates with the presence of *cis*-regulatory elements in their promoter regions.

### Full-length cloning and phylogenetic analysis of *PSY* from contrasting cultivars

**Figure 5:**
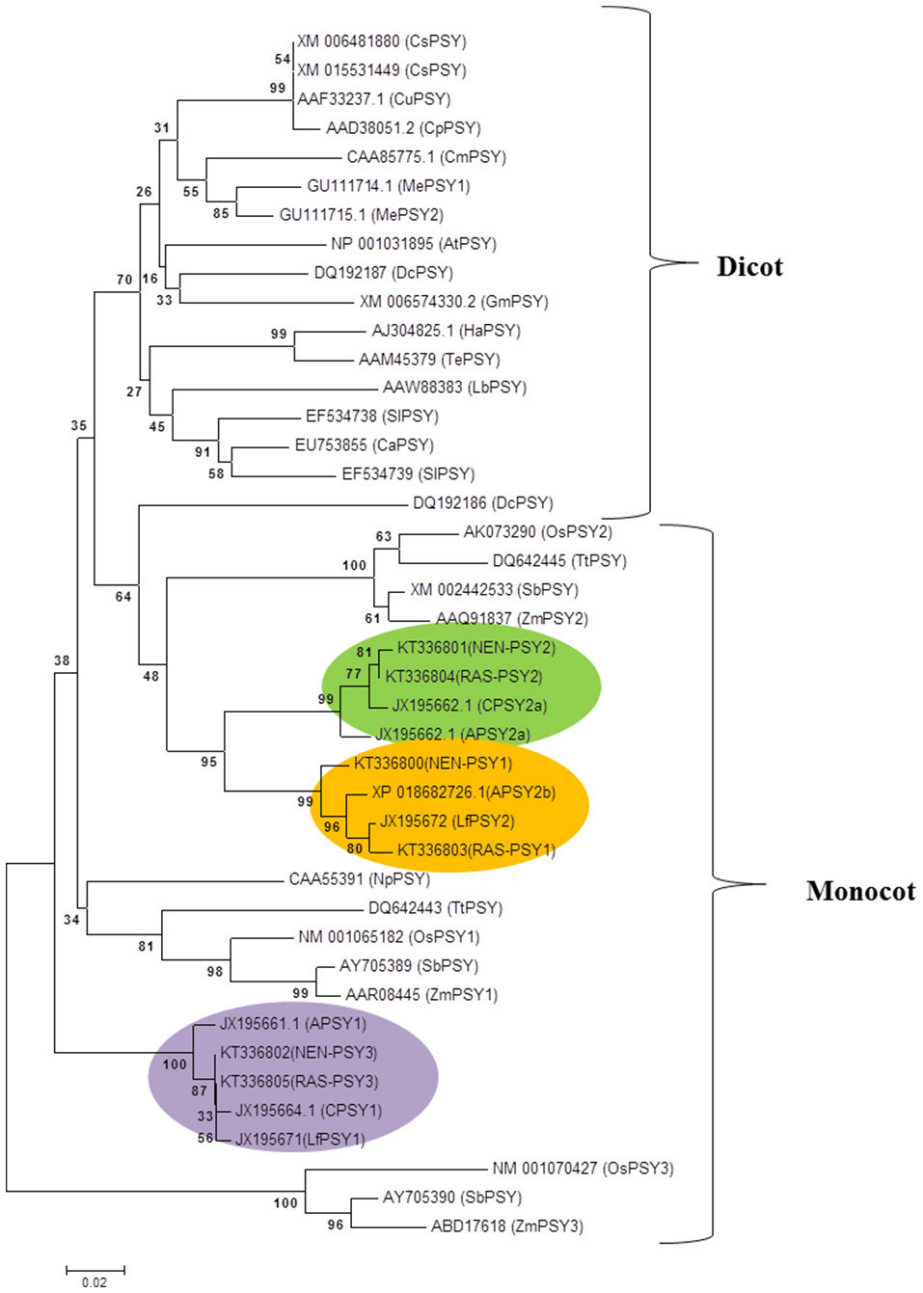
**Phylogenetic tree of PSY proteins**. Neighbor joining based phylogenetic tree of the deduced PSY protein sequences of Nendran (NEN-PSY1 - 3) and Rasthali (RAS-PSY1 - 3) inferred with different plant species. The tree was bootstrapped to 10,000 trials, and bootstrapping values are shown at each node of the rooted rectangular cladogram. The GenBank accession numbers for different PSY sequences were given in the Supplementary Table S6.

Full-length gene sequences of Nendran (*NEN-PSY1, NEN-PSY2*, and *NEN-PSY3*), and Rasthali (*RAS-PSY1, RAS-PSY2*, and *RAS-PSY3*) were amplified for characterization. The sequencing analysis revealed that all the homologs of *PSY* in both the cultivars shares considerable (≥95%) homology at both nucleotide and protein levels. The phylogenetic relationship of MaPSY proteins with known banana and other plant species was constructed using neighbor joining method. Three MaPSY identified from each of Rasthali (RAS-PSY1, RAS-PSY2, and RAS-PSY3), and Nendran (NEN-PSY1, NEN-PSY2, and NEN-PSY3) showed general trend of clustering with other monocots (Figure 5). MaPSY homologs shared three distinct clades with other known banana PSY sequences. RAS-PSY1 and NEN-PSY1 were grouped into a clade with PSY2 proteins of Asupina (APSY2b) and Ladyfinger (LfPSY2). On the other hand, RAS-PSY2 and NEN-PSY2 grouped in a clade with PSY2a proteins of Asupina and Cavendish banana varieties. RAS-PSY3 and NEN-PSY3 were clustered in a different clade with PSY1 proteins of Asupina, Cavendish and Ladyfinger.

### Motif analysis for *MaPSY* functional activity

**Figure 6:**
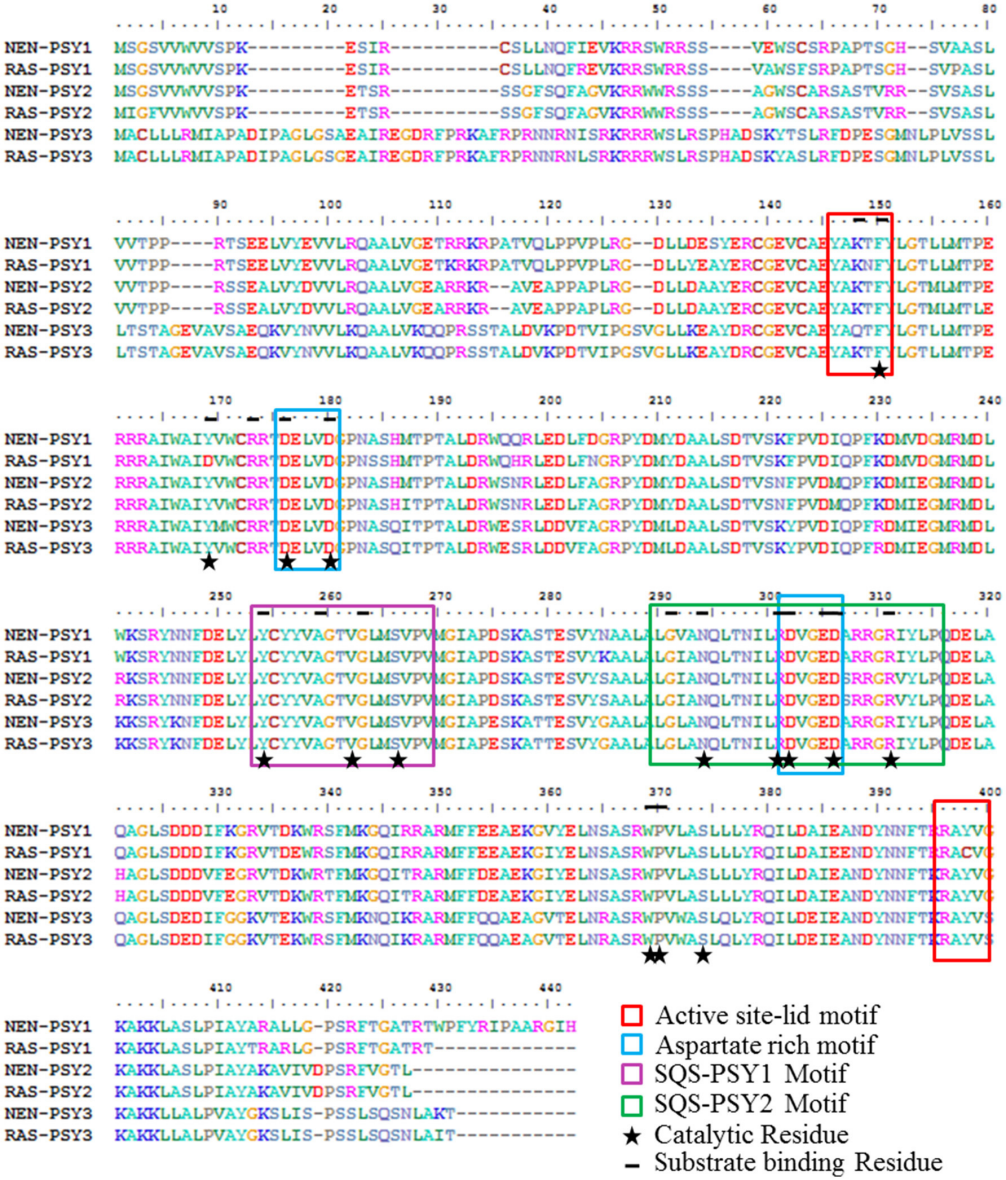
**Analysis of banana PSY protein sequences**. Multiple sequence alignment of putative protein sequences of PSY from Rasthali (RAS-PSY1 - 3) and Nendran (NEN-PSY1 - 3) cultivars.

The deduced amino acid sequences of Nendran (NEN-PSY1-3) and Rasthali (RAS-PSY1-3) were compared to determine if differences in sequences could account for the differences in fruit carotenoids content between these two cultivars of banana (Figure 6). All PSY proteins contain a predicted chloroplast transient peptide at the N-terminal region. Conserved domain analysis revealed that all the six MaPSY proteins belong to class 1 superfamily of isoprenoid biosynthesis enzymes containing the conserved trans-isoprenyl diphosphate synthases, head-to-head (trans-IPPS_HH) domain. This domain has the binding sites for substrate and cofactor that catalyzes the production of phytoene by condensation of two molecules of GGPP (Geranyl geranyl pyrophosphate). ScanProsite analysis showed the presence of squalene/phytoene synthase signatures 1 and 2 (SQS-PSY 1 and 2) in all MaPSY proteins for PSY activity. The SQS-PSY1 motif (YCyyVAGTVGlmSvpV) and aspartate-rich motifs (ARM) (DELVD and DVGED) were found conserved in each PSY protein of Nendran and Rasthali (Figure 6). This observation was further confirmed by alignments with PSY sequences of several other plant species (Supplementary Figure S4). We noted that PSY1 of both the cultivars were 95% similar and differed by a total of 19 amino acid residues which included 4 in the transit peptide and 15 in the mature protein. Among these amino acid substitutions, four located in evolutionarily conserved domains of the mature protein. The substitutions from threonine to asparagine (N149T) and tyrosine to cysteine (C398Y) in the active site lid motifs (YAKTF and RAYV), valine to isoleucine (I292V) in the SQS-PSY2 (LGianQltNIlRDVgeDarrgRiYlP), and the tyrosine to aspartic acid (D169Y) in the GGPP substrate binding pocket were detected in the RAS-PSY1 (Figure 6). PSY2 and PSY3 of both the cultivars showed nearly 99% similarities and differed by 5 and 6 amino acids, respectively. The alanine (A307) and isoleucine (I312) residues were present in SQS-PSY2 motif of the PSY1 while these were changed by serine (S307A) and valine (V312I) in PSY2 of both the cultivars. The changes of lysine to glutamine (Q148K) in active site lid-motif and isoleucine to leucine (L292I) in SQS-PSY2 motif were found in the NEN-PSY3 protein (Figure 6).

### Functional complementation analysis

**Figure 7:**
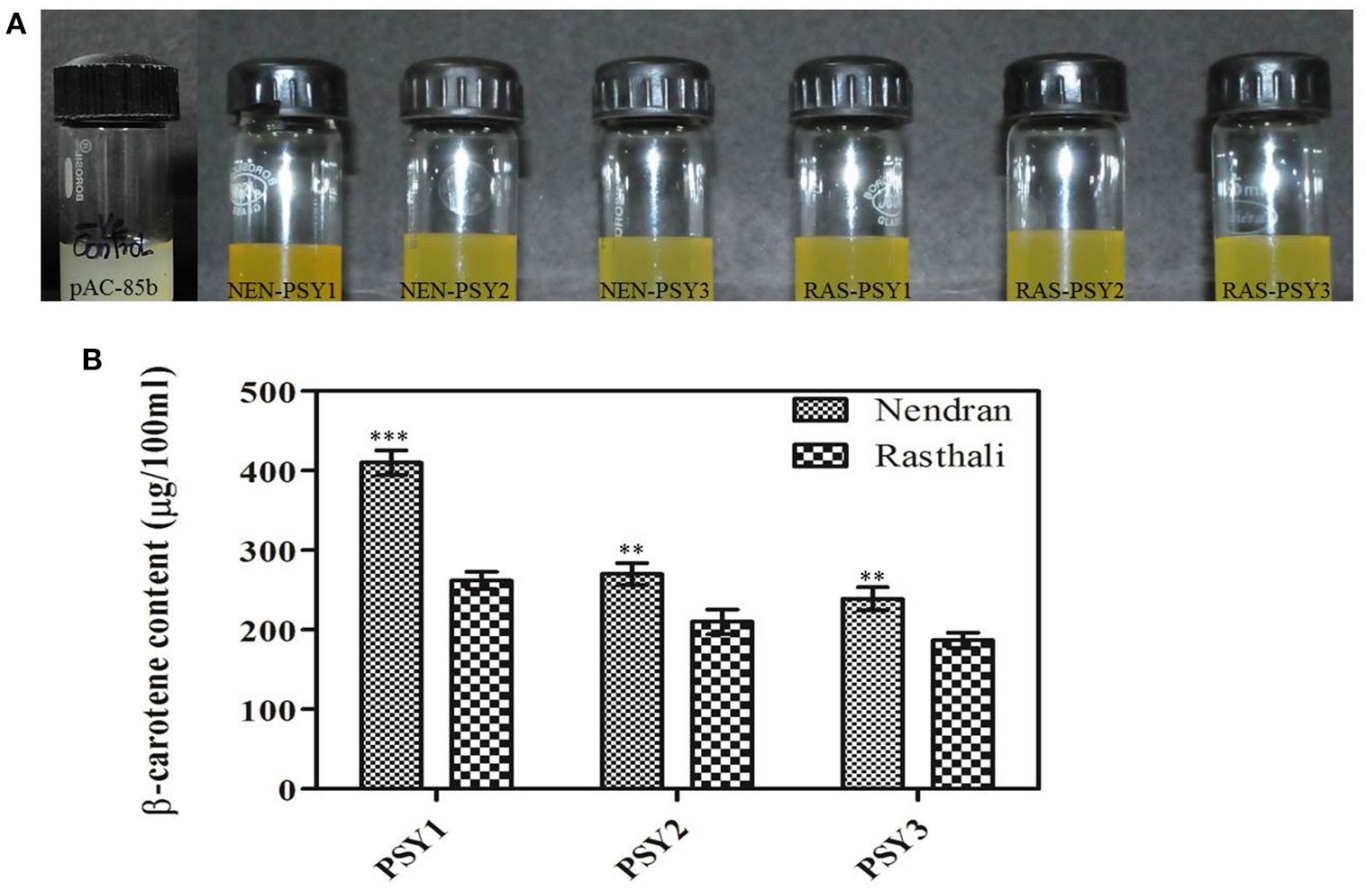
**Functional complementation analysis. (A)** Color phenotype of *E. coli* cultures consisting of pAC-85b (control) and complemented with plasmids containing *PSY* homologs (*NEN-PSY1-3* and *RAS-PSY1-3*). **(B)** Concentration of β-carotene in *E. coli* cells after complementation with plasmids pAC-85b and pTrc containing *PSY1-3* of Nendran and Rasthali. Bars denote mean of β-carotene content ± SD. Statistical significance was checked at *P* ≤ 0.05–0.001 (^**^ significant; ^***^ highly significant) with respect to the Rasthali (PSY1-3).

To determine whether the six MaPSY (NEN-PSY1, NEN-PSY2, NEN-PSY3, RAS-PSY1, RAS-PSY2, and RAS-PSY3) proteins have functional activity, the ORF of each gene was cloned into the pTrc plasmid and co-transformed along with the pAC-85b into *E. coli* TOP10F. The visual observation suggested that co-transformed cells accumulated intense yellow color as compared to the control vector pAC-85b (no color) (Figure 7A), indicating that all six MaPSY accelerate the accumulation of β-carotene but with different levels of enzymatic activity. The pigments accumulated in the transformed *E. coli* cells were extracted and analyzed by HPLC. The expected product, β-carotene was confirmed by matching spectra and retention time. The highest β-carotene content was found in the bacteria transformed with pTrc-NEN-PSY1 (410 ± 15.45 μg/100 ml culture) while the lowest was recorded with pTrc-RAS-PSY3 (187 ± 9.59 μg/100 ml culture) (Figure 7B). The β-carotene was also not detected in individually transformed pTrc-NEN-PSY1–3 and pTrc-RAS-PSY1–3 plasmid controls. These results established that all six MaPSY proteins were enzymatically functional and could accelerate the biosynthesis of β-carotene at different levels.

## Discussion

Banana is a lucrative target to be developed as a rich source of dietary carotenoid with improved agronomic traits through intervention of genetic tools. The role of PSY in the carotenoid biosynthesis (Sandmann et al., 2006; Cazzonelli and Pogson, 2010; Grassi et al., 2013; Fu et al., 2014) and stress tolerance (Li et al., 2008; Welsch et al., 2008; Arango et al., 2010) has been studied in several plants species. Therefore, an insight into the *PSY* genes involved in carotenoid biosynthesis and stress management in banana is essential for strategically selecting suitable homolog that could be utilized in the precise metabolic engineering program. In the present study, we analyzed the expression pattern of *MaPSY* homologs in different vegetative and fruit tissues. The expression of *MaPSY* in response to different environmental stimuli was also correlated with the presence of *cis*-acting elements in their promoter. The correlation between the levels of expression of *MaPSY* genes and the carotenoid content was examined to predict the homolog likely to elevate β-carotene in banana fruit pulp.

The homology and gene structure analyses suggested that the identified *MaPSY* genes have functional homologs of closely related monocots and arabidopsis. The multiple paralogs for *PSY* were found at various locations on different chromosomes in the banana genome (Supplementary Table S4). The existence of gene paralogs in banana is mainly attributed to duplication events during evolution and suggests distinct as well as overlapping roles (D'Hont et al., 2012). The banana lineage, after separation from the common ancestors of banana and poales might have experienced three whole genome duplication events, as opposed to the two in the case of poales lineage (Pandey et al., 2016).

Our phytochemical screening of different cultivars suggested that Nendran and Rasthali are the contrasting cultivars in terms of β carotene in fruit pulp (Table 1). The deep-yellow color observed in the ripe pulp of Nendran also suggested high level accumulation of carotenoids. The regulatory mechanisms leading to carotenoid synthesis and accumulation can be affected by transcriptional and/or post-transcriptional levels. The carotenoid profiling and gene expression analysis in fruit-peel and -pulp at two developmental stages (unripe and ripe) presented here gave information into transcriptional regulation of carotenoid biosynthesis in banana. The ripening induced biosynthesis of carotenoid was observed in pulp tissue in both the cultivars. However, such pattern was not noticed in the peel. Hence, their regulation in different tissues was controlled by different developmental cues. Numerous reports have highlighted antioxidant property of carotenoids (El-Agamey et al., 2004; Das and Roychoudhury, 2014; Aquino et al., 2016; García-Ruiz et al., 2017). Radical scavenging assay (inhibition of DPPH) showed ripe pulp extract has significantly higher antioxidant activity as compared to the unripe pulp. This observation is in agreement with Aquino et al. (2016). Our results suggested that the content of carotenoids in ripe pulp of Nendran is directly correlated with higher antioxidant activity (Table 4). Transcriptional regulation is an important mode to control carotenoid biosynthesis in banana fruit, as noticed by us and also in agreement with previous studies on other fruit crops such as watermelon (Grassi et al., 2013), grape (Young et al., 2012), and tomato (Giorio et al., 2008; Smita et al., 2013) where carotenoid content increases during ripening stage. The *PSY* encoding enzymatic step has been characterized as the first committed and rate-limiting step toward carotenoid biosynthesis (Bouvier et al., 2005; Sandmann et al., 2006; Cazzonelli and Pogson, 2010; Fu et al., 2014; Liu et al., 2014). Differential transcript expression of the three *PSY* genes in pulp of Nendran and Rasthali may explain the difference in carotenoids content in these two cultivars. The expression analysis revealed that *PSY1* was highly expressed in the ripe pulp of Nendran. This could be responsible for high carotenoids accumulation similar to the findings of Giorio et al. (2008) who reported the same in tomato ripening.

**Table 4:** **Measurement of antioxidant activity in extracts prepared from pulp of Nendran and Rasthali**.

	**DPPH radical scavenging activity (IC**_**50**_**)**	
	**Unripe pulp**	**Ripe pulp**
Rasthali	192.50 ± 3.53	167.62 ± 4.91
Nendran	135.91 ± 12.49^^**^^	112.90 ± 4.10^^***^^

Statistical significant was checked at p ≤ 0.05–0.0001 (

** *p ≤ 0.001*,

*** *p ≤ 0.0001) with respect to the Rasthali*.

*IC_50_, the concentration of extracts (μg/mL) causing 50% inhibition of DPPH radical*.

The expression analysis in different tissues indicated that *MaPSY* homologs were expressed at varying levels which suggest their tissue specific role in banana. The similar trend of *PSY* expression was reported in other plant species (Grassi et al., 2013; Smita et al., 2013). The up-regulation in expression of *MaPSY1* in developmental leaf stages suggests its role in growth and development.

C*is-*acting elements known to participate in various functions in promoter region of genes suggest that they could be coordinately regulated by multiple transcription factors and appears to be modulated by multiple exogenous and endogenous factors (Pandey et al., 2016). The promoter analysis indicated that the light responsive elements such as AE box and ATCT motif were exclusively absent in *MaPSY2* that is correlated with a positive modulation in the absence of light. Such up-regulation of *MaPSY2* expression suggests it salient role during the dark condition as earlier reported for the Maize *PSY1* (Li et al., 2008).

ABA is a *cis-*carotenoid metabolite that can alter the production of carotenoids (Kachanovsky et al., 2012). We found that ABRE, myb binding site (MBS) and G-box are responsible for ABA response were located in the promoter region of *MaPSY2* suggested it possible role in defense mechanism. Enhanced expression of *MaPSY1* and *MaPSY2* genes was observed during ABA treatment, although ABRE element was absent in proximal promoter of *PSY1*. This might be due to ABA induced production of ethylene (Zhang et al., 2009a,b) and many other metabolites (Jeong et al., 2004; Giribaldi et al., 2010) those have significant role in fruit ripening. Moreover, ABA is a *cis-*carotenoid metabolite can alter the production of carotenoids which are precursors for the ABA biosynthesis (Kachanovsky et al., 2012).

SA protects the chloroplast membrane and enhances the antioxidant capacity of the plant (Huang et al., 2016) under stresses. Furthermore, MeJA has been reported to enhance the β-carotene content in broccoli sprouts (Natella et al., 2016) and in mango (Muengkaew et al., 2016). Impositions of SA and MeJA on banana plantlets showed positive modulation of *MaPSY1* with respect to other genes, delineating its role in plant development, physiology and antioxidant defense mechanism.

The functional protein encoded by *PSY* should include the Lid, ARM, and SQS-PSY evolutionarily conserved domains which are responsible for substrate binding and the catalytic activity (Mlalazi et al., 2012; López-Emparán et al., 2014). Mutation of critical amino acid residues in PSY changes the functionality of the mature protein (Howitt et al., 2009; Welsch et al., 2010; Gady et al., 2012; Mlalazi et al., 2012; Shumskaya et al., 2012; Nogueira et al., 2013), thus impacting on carotenoid biosynthesis.

All three PSY1-3 sequences of Nendran and Rasthali contain these conserved domains. However, in-depth sequence analysis revealed that NEN-PSY1, NEN-PSY2 and NEN-PSY3 are differed from RAS-PSY1, RAS-PSY2, and RAS-PSY3, in 19, five and six amino acids, respectively. These differences may play an important role in the carotenoid content between the two cultivars. Threonine and tyrosine are reported to be more conserved amino acids in functional active domains of PSY in several plant systems (Supplementary Figure S4). The change in active site lid motif threonine to asparagine (N149T) and tyrosine to cysteine (C398Y), SQS-PSY2 motif valine to isoleucine (I292V) and the replacement of tyrosine residue with aspartic acid (D169Y) involved in the catalytic activity as well as substrate binding might result in alteration of catalytic activity and substrate binding efficiency of the enzyme encoded by *RAS-PSY1*. Substitutions in PSY2 and PSY3 in their SQS-PSY2 and active site lid motif may also lead to variation in the carotenoid content found in Nendran and Rasthali. The similar observation was noticed by Mlalazi et al. (2012) where the differences in the 10 amino acid sequences of Cavendish and Asupina PSY2a showed upto 20-fold higher β-carotene content in Asupina fruit. Moreover, differences in amino acids at different positions in the putative peptide may affect the structural properties of protein by altering interactions between the residues adjacent to each other. However, the impact of different amino acid substitutions on PSY activity requires further characterization.

We analyzed and compared the functional activities of all six MaPSY using a bacterial complementation system. Similar system has already been used to demonstrate the function of different carotenoid pathway genes from hazel (Wang et al., 2007), papaya (Devitt et al., 2010), jatropha (Lin et al., 2010), grapevine (Young et al., 2012), and loquat (Fu et al., 2014). We observed significantly higher activity with NEN-PSY1 compared to the other homologs suggesting its role in the carotenoid biosynthesis (Figure 5). Differential accumulation of β-carotene in bacterial system transformed with *PSY* could be affected by the variety of factors including catalytic activity, protein localization, stability, folding, solubility and/or the differences in amino acid sequence found between the different homologs. Hence, it would be needed to confirm the functional activity of banana PSY in plant. Further, it also remains to be seen if carotenoid degrading enzymes such as carotenoid cleavage dioxygenases (CCDs), β-ring hydroxylases (BCHs) and 9-cis carotenoid cleavage dioxygenases (NCEDs) play any critical role in carotenoid accumulation in banana fruit.

## Accession numbers

**Sequence data from this article have been deposited in the GenBank data libraries under accession numbers:** NEN-PSY1 (KT336800), NEN-PSY2 (KT336801), NEN-PSY3 (KT336803), RAS-PSY1 (KT336804), RAS-PSY2 (KT336805), and RAS-PSY3 (KT336807).

## Author contributions

NK, AP, and ST conceived the idea and designed research experiments. ST supervised the research. NK, AP, SS, PK, and PP performed the experiments related to plants. NK, AP, PK, and AK performed phytochemical analysis. NK and PA performed antioxidant activity. NK, AP, and SM performed bioinformatics analysis. NK, AP, and ST analyzed data, prepared the figures and wrote the manuscript.

### Conflict of interest statement

The authors declare that the research was conducted in the absence of any commercial or financial relationships that could be construed as a potential conflict of interest.

## Supplementary material

Supplementary Figure S1 **Schematic representation of pathways for biosynthesis of MVA, MEP and carotenoids in higher plants**. Enzymatic reactions are represented by arrows; dashed lines represent multiple enzymatic steps. Enzymes: HMGS, 3-hydroxy-3-methylglutaryl-CoA synthase; HMGR, 3-hydroxy3-methylglutaryl-CoA reductase; DXS, 1- deoxy-D-xylulose-5-phosphate synthase; DXR, 1- deoxy-D-xylulose-5-phosphate reductase; HDR, 1-hydroxy-2-methyl-2-(E)-butenyl-4diphosphate reductase; GGPP synthase, geranylgeranyl diphosphate synthase; PSY, phytoene synthase; PDS, phytoene desaturase; ZDS, ζ-carotene desaturase; LCYB, lycopene β-cyclase; LCYE, lycopene ε-cyclase; CRTISO, carotenoid isomerase; BCH, β-ring hydroxylase, ECH, ε-ring hydroxylase. Compounds: HMG-CoA, 3-hydroxy-3methylglutaryl-CoA; MVA, mevalonic acid; GAP, glyceraldehyde-3-phosphate; DOXP, 1deoxy-D-xylulose-5-phosphate; MEP, 2-C-methyl-D-erythritol 4-phosphate; HMBPP, 1hydroxy-2-methyl-2-(E)-butenyl-4-diphosphate; IPP, isopentenyl diphosphate; DMAPP, dimethylallyl diphosphate; GGPP, geranylgeranyl diphosphate.

Supplementary Figure S2 **Representative HPLC profiles illustrating the accumulation of carotenoids in fruit-pulp of banana**. Peaks in chromatograms are representing 1: lutein, 2: zeaxanthin, 3: α-carotene, 4: β-carotene and 5: lycopene. Extract was separated with HPLC coupled with UV and PDA detector.

Supplementary Figure S3 **Representative HPLC profiles illustrating the accumulation of carotenoids in fruit-peel of banana**. Peaks in chromatograms are representing 1: lutein, 2: zeaxanthin, 3: α-carotene, 4: β-carotene and 5: lycopene. Extract was separated with HPLC coupled with UV and PDA detector.

Supplementary Figure S4 **Analysis of PSY protein sequences**. Multiple sequence alignment of putative protein sequences of PSY1 of Rasthali and Nendran along with different monocots PSY sequences. The GenBank accession numbers for different PSY sequences were given in the Supplementary Table S6.

Supplementary Table S1 **List of the primers used in the present study**.

Supplementary Table S2 **Percentage identity of the predicted protein sequence of ***MaPSY*****.

Supplementary Table S3 **Gene families and the exons and introns numbers in ***MaPSY*** homologs**.

Supplementary Table S4 **Structural features of three ***PSY*** homologs in banana**.

Supplementary Table S5 *****Cis***-regulatory elements in the promoter of ***MaPSY***genes**.

Supplementary Table S6 **PSY protein sequences used for phylogenetic tree analysis in Figure 5**.
