# Supplementary figures and images for: Regulation of Banana Phytoene Synthase (MaPSY) Expression, Characterization and Their Modulation under Various Abiotic Stress Conditions
Source: Front Plant Sci. 2017 Apr 3;8:462. doi: 10.3389/fpls.2017.00462 (PMC5377061; doi:10.3389/fpls.2017.00462)

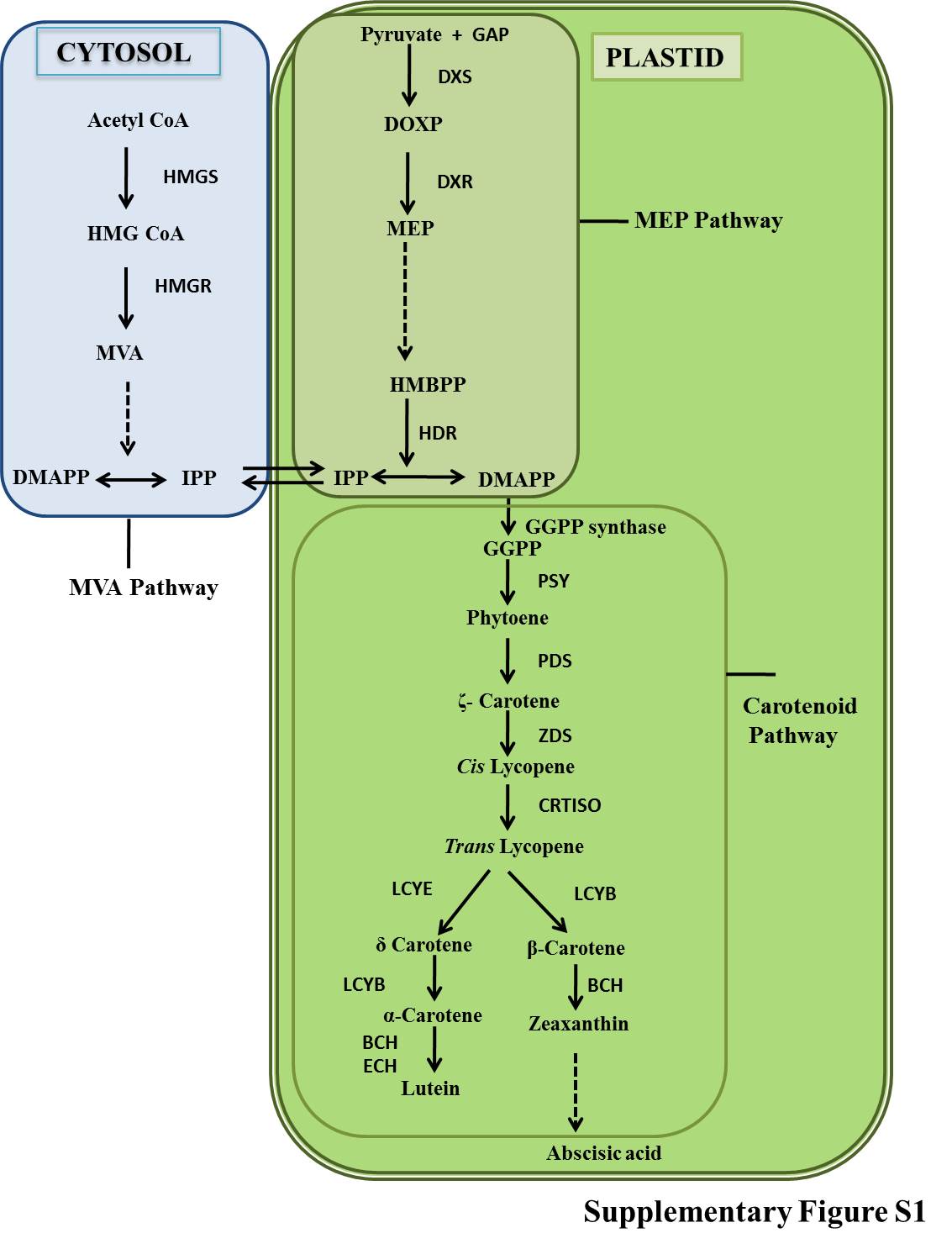

Supplement: Supplementary Figure S1 — Schematic representation of pathways for biosynthesis of MVA, MEP and carotenoids in higher plants. Enzymatic reactions are represented by arrows; dashed lines represent multiple enzymatic steps. Enzymes: HMGS, 3-hydroxy-3-methylglutaryl-CoA synthase; HMGR, 3-hydroxy3-methylglutaryl-CoA reductase; DXS, 1- deoxy-D-xylulose-5-phosphate synthase; DXR, 1- deoxy-D-xylulose-5-phosphate reductase; HDR, 1-hydroxy-2-methyl-2-(E)-butenyl-4diphosphate reductase; GGPP synthase, geranylgeranyl diphosphate synthase; PSY, phytoene synthase; PDS, phytoene desaturase; ZDS, ζ-carotene desaturase; LCYB, lycopene β-cyclase; LCYE, lycopene ε-cyclase; CRTISO, carotenoid isomerase; BCH, β-ring hydroxylase, ECH, ε-ring hydroxylase. Compounds: HMG-CoA, 3-hydroxy-3methylglutaryl-CoA; MVA, mevalonic acid; GAP, glyceraldehyde-3-phosphate; DOXP, 1deoxy-D-xylulose-5-phosphate; MEP, 2-C-methyl-D-erythritol 4-phosphate; HMBPP, 1hydroxy-2-methyl-2-(E)-butenyl-4-diphosphate; IPP, isopentenyl diphosphate; DMAPP, dimethylallyl diphosphate; GGPP, geranylgeranyl diphosphate. [file Image1.JPEG]

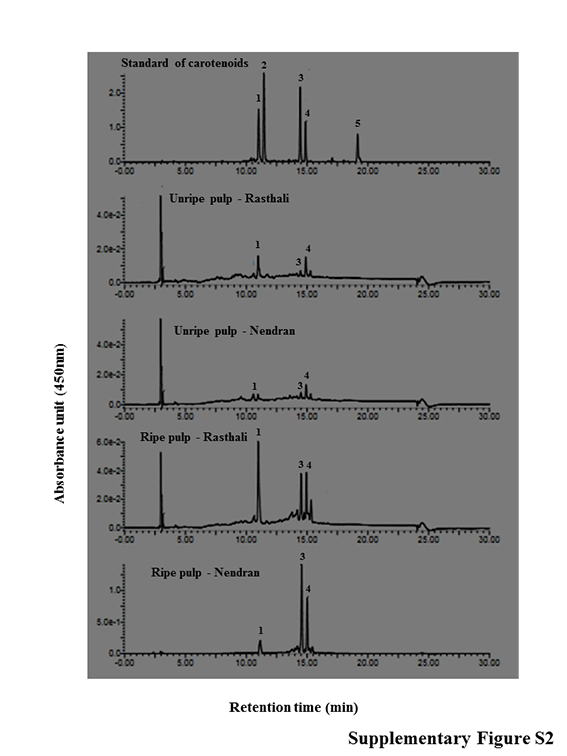

Supplement: Supplementary Figure S2 — Representative HPLC profiles illustrating the accumulation of carotenoids in fruit-pulp of banana. Peaks in chromatograms are representing 1: lutein, 2: zeaxanthin, 3: α-carotene, 4: β-carotene and 5: lycopene. Extract was separated with HPLC coupled with UV and PDA detector. [file Image2.TIF]

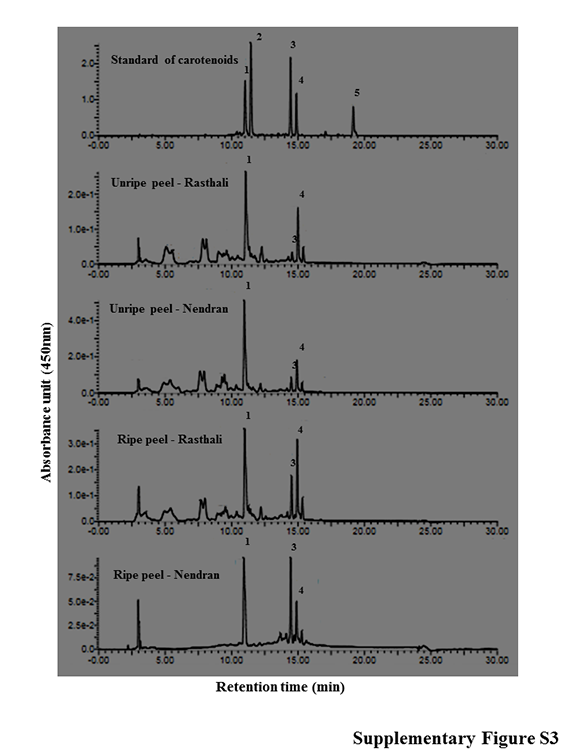

Supplement: Supplementary Figure S3 — Representative HPLC profiles illustrating the accumulation of carotenoids in fruit-peel of banana. Peaks in chromatograms are representing 1: lutein, 2: zeaxanthin, 3: α-carotene, 4: β-carotene and 5: lycopene. Extract was separated with HPLC coupled with UV and PDA detector. [file Image3.TIF]

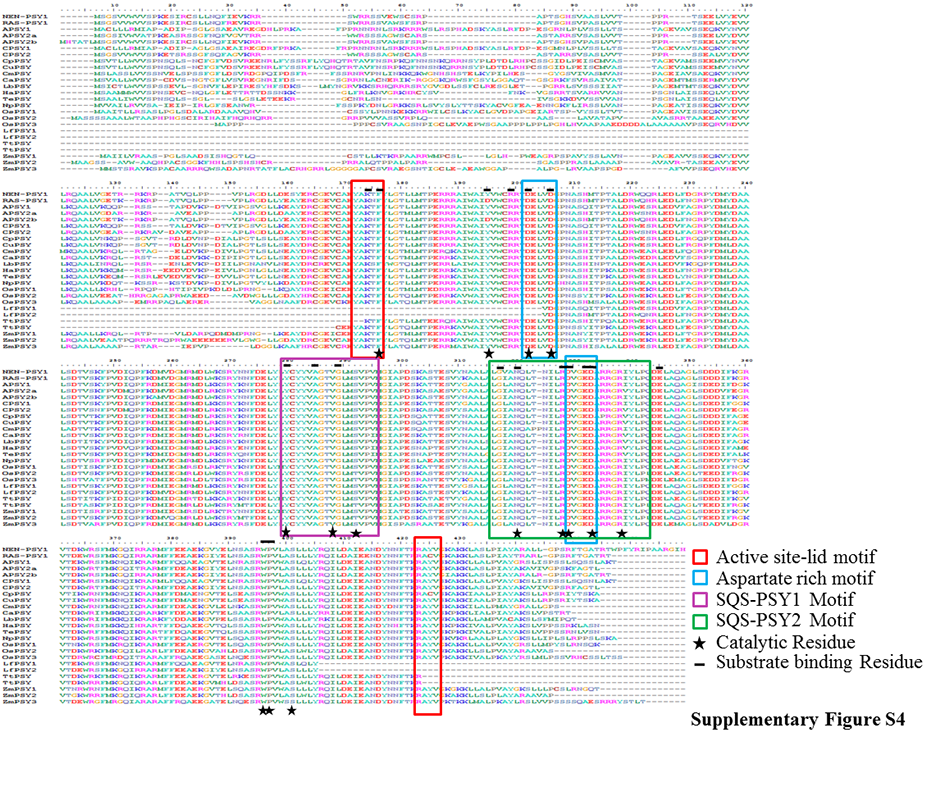

Supplement: Supplementary Figure S4 — Analysis of PSY protein sequences. Multiple sequence alignment of putative protein sequences of PSY1 of Rasthali and Nendran along with different monocots PSY sequences. The GenBank accession numbers for different PSY sequences were given in the Supplementary Table S6. [file Image4.TIF]
