# Supplementary material for: Regulation of Banana Phytoene Synthase (MaPSY) Expression, Characterization and Their Modulation under Various Abiotic Stress Conditions
Source: Front Plant Sci. 2017 Apr 3;8:462. doi: 10.3389/fpls.2017.00462 (PMC5377061; doi:10.3389/fpls.2017.00462)
Supplement: Supplementary Table S1 — List of the primers used in the present study. [file Table1.DOCX]

**Supplementary Table 1. List of the primers used in the present study.**

| **Primer name** | **Forward Sequence (5’ to 3’)** | **Reverse Sequence (5’ to 3’)** | **Purpose** |
| --- | --- | --- | --- |
| *MaPSY1* | TTGCTACGGATGATTGCTCC | TTCCTGTTGTTCCTTGGACG | Real-time PCR |
| *MaPSY2* | ACATGATCGAAGGAATGCGA | GCTCATCAGTCCAACAGTTCC | Real-time PCR |
| *MaPSY3* | ATGCTAGGAGGGGGAGAATC | GCTCGTATATGCCCTTCTCG | Real-time PCR |
| *MaPSY1* | GGAAGATCTATGTCTGGCTCTG  TTGTCTGGGTT | ATAAGAATGCGGCCGCTTATGTCCTTGTTGCTCCTGTAAACC | Cloning |
| *MaPSY2* | GGAAGATCTATGTCTGGCTCTG  TTGTTTGGGTTG | ATAAGAATGCGGCCGCTTATAGTGTTCCTACAAATCTTGAAGGGC | Cloning |
| *MaPSY3* | GGAAGATCTATGGCGTGCCTGT  TGCTACGGATG | ATAAGAATGCGGCCGCTCATGTTTTTGCTAAGTTTGACTGGCTCAG | Cloning |
