# Supplementary material for: Regulation of Banana Phytoene Synthase (MaPSY) Expression, Characterization and Their Modulation under Various Abiotic Stress Conditions
Source: Front Plant Sci. 2017 Apr 3;8:462. doi: 10.3389/fpls.2017.00462 (PMC5377061; doi:10.3389/fpls.2017.00462)
Supplement: Supplementary Table S2 — Percentage identity of the predicted protein sequence of MaPSY. [file Table2.DOCX]

**Supplementary Table 2.Percentage identity of the predicted protein sequence of *MaPSY*.**

| **GENE** | ***M.acuminata*** | ***O. sativa*** | ***Z. mays*** | ***A. thaliana*** |
| --- | --- | --- | --- | --- |
| ***MaPSY1*** | GSMUA_Achr6P31560_001 | LOC_Os06g51290.1  71%  LOC_Os06g51290.4  70%  LOC_Os09g38320.1  72%  LOC_Os12g43130.1  80% | ZM08G31920  69%  ZM06G09680  66%  NP_001108124  67%  ABD17618.1  71% | AT5G17230.1  73% |
| ***MaPSY2*** | GSMUA_Achr9P10050_001 | LOC_Os06g51290.1  64%  LOC_Os06g51290.4  55%  LOC_Os09g38320.1  56%  LOC_Os12g43130.1  67% | ZM08G31920  78%  ZM06G09680  70%  NP_001108124  71%  ABD17618.1  73% | AT5G17230.1  74% |
| ***MaPSY3*** | GSMUA_AchrUn_randomP09240_001 | LOC_Os06g51290.1  74%  LOC_Os06g51290.4  73%  LOC_Os09g38320.1  72%  LOC_Os12g43130.1  74% | ZM08G31920  74%  ZM06G09680  78%  NP_001108124  79%  ABD17618.1  71% | AT5G17230.1  81% |
