# Supplementary material for: Regulation of Banana Phytoene Synthase (MaPSY) Expression, Characterization and Their Modulation under Various Abiotic Stress Conditions
Source: Front Plant Sci. 2017 Apr 3;8:462. doi: 10.3389/fpls.2017.00462 (PMC5377061; doi:10.3389/fpls.2017.00462)
Supplement: Supplementary Table S3 — Gene families and the exons and introns numbers in MaPSY homologs. [file Table3.DOCX]

**Supplementary Table 3.Gene families and the exons and introns numbers in *MaPSY* homologs.**

| **Gene** | **Gene family** | **Sub family** | ***M.acuminata*** | ***O.sativa*** | ***Z.mays*** | ***A.thaliana*** |
| --- | --- | --- | --- | --- | --- | --- |
| ***MaPSY*** | HOM005200 | ORTHO004910 | GSMUA_Achr6P31560_001 **6(5)** | LOC_Os06g51290.1 **6(5)** | ZM08G31920 **6(5)** | AT5G17230.1 **1(0)** |
|  |  |  | GSMUA_Achr9P10050_001 **6(5)** | LOC_Os06g51290.4 **6(5)** | ZM06G09680 **6(5)** |  |
|  |  |  | GSMUA_AchrUn_randomP09240_001 **6(5)** | LOC_Os09g38320.1 **6(5)** | NP_001108124**6(5)** |  |
|  |  |  |  | LOC_Os12g43130.1 **6(5)** | ABD17618.1**6(5)** |  |
