# Supplementary material for: Regulation of Banana Phytoene Synthase (MaPSY) Expression, Characterization and Their Modulation under Various Abiotic Stress Conditions
Source: Front Plant Sci. 2017 Apr 3;8:462. doi: 10.3389/fpls.2017.00462 (PMC5377061; doi:10.3389/fpls.2017.00462)
Supplement: Supplementary Table S4 — Structural features of three PSY homologs in banana. [file Table4.DOCX]

**Supplementary Table 4.Structural features of three *PSY* homologs in banana.**

| S. No. | **Gene name** | **Gene ID** | **Chromosome position** | **Genomic coordinate**  **Start (bp) End (bp)** | **Proteinlength (amino acid)** | **Predicted PI** | **Molecular weight (kDa)** | **Subcellular localization** | **Transmembrane helix** (**TMH)** |
| --- | --- | --- | --- | --- | --- | --- | --- | --- | --- |
| 1 | ***MaPSY1*** | GSMUA_Achr6P31560_001 | 6 | 31294343 31298625 | 397 | 9.22 | 45.03 | Chloroplast | 0 |
| 2 | ***MaPSY2*** | GSMUA_Achr9P10050_001 | 9 | 6467097 6469206 | 397 | 9.12 | 44.35 | Chloroplast | 1 |
| 3 | ***MaPSY3*** | GSMUA_AchrUn_randomP09240_001 | Random | 42933243 42935315 | 426 | 9.38 | 47.76 | Chloroplast | 0 |
