# Supplementary material for: Regulation of Banana Phytoene Synthase (MaPSY) Expression, Characterization and Their Modulation under Various Abiotic Stress Conditions
Source: Front Plant Sci. 2017 Apr 3;8:462. doi: 10.3389/fpls.2017.00462 (PMC5377061; doi:10.3389/fpls.2017.00462)
Supplement: Supplementary Table S5 — Cis-regulatory elements in the promoter of MaPSYgenes. [file Table5.DOCX]

**Supplementary Table 5.*Cis*-regulatory elements in the promoter of *MaPSY*genes.**

| **Gene Name** | **Motifs related to growth and development** | **Motifs related to light response** | **Motifs related to stress response** |
| --- | --- | --- | --- |
| ***MaPSY1*** | AC-II, GCN4_motif, O2-site, Skn-1_motif, | AE-box, ATCT-motif, Box 4, Box I, CATT-motif, GATA-motif, I-box, Sp1, as-2-box, chs-CMA1a | Box-W1, CGTCA-motif, HSE, MBS, TCA-element, TGACG-motif, W box, box E |
| ***MaPSY2*** | GCN4_motif, Skn-1_motif, circadian | ACE, ATC-motif, Box 4, G-Box, G-box, MRE, Sp1, TCT-motif | ABRE, Box-W1, CGTCA-motif , C-repeat/DRE, GARE-motif, MBS, TC-rich repeats, TGACG-motif, W box, |
| ***MaPSY3*** | CCGTCC-box, Skn-1_motif, circadian | AE-box, ATCT-motif, Box 4, Box I, Box III, CATT-motif, GT1-motif, I-box, MNF1, P-box, Sp1, TCT-motif, | C-repeat/DRE, TATC-box , TC-rich repeats, TCA-element, box E |
