# Supplementary material for: Regulation of Banana Phytoene Synthase (MaPSY) Expression, Characterization and Their Modulation under Various Abiotic Stress Conditions
Source: Front Plant Sci. 2017 Apr 3;8:462. doi: 10.3389/fpls.2017.00462 (PMC5377061; doi:10.3389/fpls.2017.00462)
Supplement: Supplementary Table S6 — PSY protein sequences used for phylogenetic tree analysis in Figure 5. [file Table6.DOCX]

**Supplementary Table 6**. **PSY protein sequences used for phylogenetic tree analysis in Figure 5.**

| **Name** | **Accession number** | **Common name/Source** |
| --- | --- | --- |
| Asupina PSY1 | JX195661.1 | Banana |
| Asupina PSY2a | JX195662.1 | Banana |
| Asupina PSY2b | XP_018682726.1 | Banana |
| Cavendish PSY1 | JX195664.1 | Banana |
| Cavendish PSY2a | JX195662.1 | Banana |
| LadyfingerPSY1 | JX195671 | Banana |
| LadyfingerPSY2 | JX195672 | Banana |
| AtPSY1 | NP_001031895 | Arabidopsis |
| CpPSY | AAD38051.2 | Grape fruit |
| CuPSY | AAF33237.1 | Orange |
| CmPSY | CAA85775.1 | Melon |
| CaPSY | EU753855 | Capsicum |
| LbPSY | AAW88383 | Wolfberry |
| HaPSY | AJ304825.1 | Sunflower |
| TePSY | AAM45379 | Marigold |
| NpPSY | CAA55391 | Daffodil |
| JaponicaPSY1 | NM_001065182 | Rice |
| JaponicaPSY2 | AK073290 | Rice |
| JaponicaPSY3 | NM_001070427 | Rice |
| SbPSY1 | AY705389 | Millet |
| SbPSY2 | XM_002442533 | Millet |
| SbPSY3 | AY705390 | Millet |
| TtPSY1 | DQ642443 | Wheat |
| TtPSY2 | DQ642445 | Wheat |
| ZmPSY1 | AAR08445 | Maize |
| ZmPSY2 | AAQ91837 | Maize |
| ZmPSY3 | ABD17618 | Maize |
| DcPSY1 | DQ192186 | Carrot |
| DcPSY2 | DQ192187 | Carrot |
| SlPSY1 | EF534739 | Tomato |
| SlPSY2 | EF534738 | Tomato |
| MePSY1 | GU111714.1 | Cassava |
| MePSY2 | GU111715.1 | Cassava |
| CsPSY1 | XM_006481880 | Citrus |
| CsPSY2 | XM_015531449 | Citrus |
| GmPSY | XM_006574330.2 | Soybean |
| VvPSY | AM481588.1 | Grape |

The GenBank accession numbers for Nen-PSY1 (KT336800), Nen-PSY2 (KT336801), Nen-PSY3 (KT336802), Ras-PSY1 (KT336804), Ras-PSY2 (KT336805), Ras-PSY3 (KT336806).
